# Complete Genome Sequences of Five Zika Virus Isolates

**DOI:** 10.1128/genomeA.00377-16

**Published:** 2016-05-12

**Authors:** Jason T. Ladner, Michael R. Wiley, Karla Prieto, Chadwick Y. Yasuda, Elyse Nagle, Matthew R. Kasper, Daniel Reyes, Nikolaos Vasilakis, Vireak Heang, Scott C. Weaver, Andrew Haddow, Robert B. Tesh, Ly Sovann, Gustavo Palacios

**Affiliations:** a Center for Genome Sciences, U.S. Army Medical Research Institute of Infectious Diseases, Fort Detrick, Frederick, Maryland, USA; b U.S. Naval Medical Research Unit No. 2, Det Phnom Penh (NAMRU-2), Cambodia; c Naval Medical Research Center, Silver Spring, Maryland, USA; d Institute for Human Infections and Immunity, University of Texas Medical Branch, Galveston, Texas, USA; e Department of Pathology, University of Texas Medical Branch, Galveston, Texas, USA; f Department of Microbiology and Immunology, University of Texas Medical Branch, Galveston, Texas, USA; g Division of Virology, U.S. Army Medical Research Institute of Infectious Diseases, Fort Detrick, Frederick, Maryland, USA; h Communicable Disease Centre, Cambodian Ministry of Health, Phnom Penh, Cambodia

## Abstract

Zika virus is an emerging human pathogen of great concern due to putative links to microcephaly and Guillain-Barre syndrome. Here, we report the complete genomes, including the 5′ and 3′ untranslated regions, of five Zika virus isolates, one from the Asian lineage and four from the African lineage.

## GENOME ANNOUNCEMENT

Zika virus (ZIKV) is an emerging human pathogen of great concern due to putative links to severe birth defects (microcephaly [1]) and a rare autoimmune syndrome (Guillain-Barre [2]). The virus was first detected in a sentinel rhesus macaque in 1947 while monitoring for enzootic yellow fever virus circulation in the Zika Forest, Uganda (3). Since then, ZIKV has been detected throughout much of Sub-Saharan Africa and has spread across the Pacific Ocean to Central and South America via Southeast Asia (4, 5).

ZIKV is an arbovirus believed to be vectored mainly by mosquitoes in the genus *Aedes*. ZIKV belongs to the Spondweni serocomplex within the genus *Flavivirus* and the family *Flaviviridae*. Viruses in the genus *Flavivirus* have single-stranded positive-sense RNA genomes of ~11 kb in size (6). These genomes encode a single polyprotein flanked by 5′ and 3′ untranslated regions (UTRs).

Here, we report five complete ZIKV genomes, including the full polyprotein and the 5′ and 3′ UTRs. Four of these viruses were isolated in Africa (Senegal and Uganda) and belong to the African lineage (4). One virus (FSS13025) was isolated in Cambodia and belongs to the Asian lineage (4). All five isolates were passaged multiple times in cell culture (Table 1). Several distinctly passaged versions of strain MR-766 have been previously published, including two complete genomes (GenBank: AY632535, LC002520). A coding-complete version (i.e., missing the 5′ and 3′ UTRs [7]) of the genome was published for an earlier passage of FSS13025 (GenBank: JN860885).

**TABLE 1:** Accession numbers and source information for sequenced Zika virus isolates

Isolate	Source	Location	Date	Passage history^a^	Genome/ORF^b^ size (nucleotides)	Accession no.
FSS13025	*Homo sapiens*	Cambodia	8/8/2010	Vero ×4	10,807/10,272	KU955593
DAK AR D 41671	*Aedes taylori*	Senegal	12/14/1984	AP61 ×1, C6/36 ×1, Vero ×3	10,806/10,272	KU955595
DAK AR D 41662	*Aedes taylori*	Senegal	12/6/1984	AP61 ×1, C6/36 ×1, Vero ×3	10,806/10,272	KU955592
DAK AR D 41525	*Aedes africanus*	Senegal	11/20/1984	AP61 ×1, C6/36 ×1, Vero ×3	10,806/10,272	KU955591
MR-766	*Macaca mulatta*	Uganda	4/1947	SM ×150, Vero ×2	10,795/10,260	KU955594

a AP61, *Aedes pseudoscutellaris* cells; C6/36, *Aedes albopictus* cells; SM, suckling mouse; Vero, African green monkey kidney cells.

b ORF, open reading frame.

Isolates were sequenced at the U.S. Army Medical Research Institute of Infectious Diseases and the University of Texas Medical Branch using Illumina NextSeq/MiSeq. RNA was extracted using the Direct-zol RNA extraction kit (Zymo), converted to cDNA using SuperScript III (Invitrogen) and amplified using sequence-independent single-primer amplification (8) combined with primers for rapid amplification of cDNA ends (9). Adaptors and primers were clipped using Cutadapt version 1.21 (10), and low-quality reads/bases were filtered using Prinseq-lite version 0.20.4 (11). Reads were aligned to a reference genome (chosen separately for each isolate based on nucleotide-level similarity between preliminary *de novo* contigs and publically available ZIKV genomes) using Bowtie2 (12), duplicates were removed with Picard (http://broadinstitute.github.io/picard), and a new consensus was generated using a combination of SAMtools version 0.1.18 (13) and custom scripts. When necessary, the 5′ and 3′ UTRs were then built out beyond the reference genome using custom scripts that iterate the reference assembly process with the addition of ambiguous characters (N’s) onto the 5′ and 3′ ends of the reference.

The assembled, complete genomes (7) ranged in size from 10,795 to 10,807 nucleotides; the 5′ UTRs were 106 to 107 nucleotides and the 3′ UTRs were 428 to 429 nucleotides, respectively. The MR-766 stock we sequenced contained a 12-nucleotide in-frame deletion in the polyprotein, which it shared with AY632535, but is not present in LC002520. However, our sequence does not share the five additional single nucleotide insertions/deletions present in AY632535. All sequenced isolates contained the conserved, dinucleotide complimentary terminal sequences that are characteristic of the *Flavivirus* genus (5′ – AG … CU – 3′ [6]).

### Nucleotide sequence accession numbers.

Genome accession numbers to public databases are listed in Table 1.
